# Gemcitabine-mediated depletion of immunosuppressive dendritic cells enhances the efficacy of therapeutic vaccination

**DOI:** 10.3389/fimmu.2022.991311

**Published:** 2022-10-10

**Authors:** David Repáraz, Marta Ruiz, Leyre Silva, Belén Aparicio, Josune Egea, Elizabeth Guruceaga, Daniel Ajona, Yaiza Senent, Enrique Conde, Flor Navarro, Sergio Barace, Diego Alignani, Sandra Hervás-Stubbs, Juan José Lasarte, Diana Llopiz, Pablo Sarobe

**Affiliations:** ^1^ Centro de Investigación Médica Aplicada (CIMA), Universidad de Navarra, Pamplona, Spain; ^2^ IdiSNA, Instituto de Investigación Sanitaria de Navarra, Pamplona, Spain; ^3^ Centro de Investigación Biomédica en Red de Enfermedades Hepáticas y Digestivas (CIBEREHD), Pamplona, Spain; ^4^ Department of Biochemistry and Genetics, School of Sciences, University of Navarra, Pamplona, Spain; ^5^ Centro de Investigación Biomédica en Red de Cáncer (CIBERONC), Madrid, Spain

**Keywords:** immunosuppressive DC, antitumor therapeutic vaccination, monocyte depletion, anti-PD-1, gemcitabine

## Abstract

Vaccination using optimized strategies may increase response rates to immune checkpoint inhibitors (ICI) in some tumors. To enhance vaccine potency and improve thus responses to ICI, we analyzed the gene expression profile of an immunosuppressive dendritic cell (DC) population induced during vaccination, with the goal of identifying druggable inhibitory mechanisms. RNAseq studies revealed targetable genes, but their inhibition did not result in improved vaccines. However, we proved that immunosuppressive DC had a monocytic origin. Thus, monocyte depletion by gemcitabine administration reduced the generation of these DC and increased vaccine-induced immunity, which rejected about 20% of LLC-OVA and B16-OVA tumors, which are non-responders to anti-PD-1. This improved efficacy was associated with higher tumor T-cell infiltration and overexpression of PD-1/PD-L1. Therefore, the combination of vaccine + gemcitabine with anti-PD-1 was superior to anti-PD-1 monotherapy in both models. B16-OVA tumors benefited from a synergistic effect, reaching 75% of tumor rejection, but higher levels of exhausted T-cells in LLC-OVA tumors co-expressing PD-1, LAG3 and TIM3 precluded similar levels of efficacy. Our results indicate that gemcitabine is a suitable combination therapy with vaccines aimed at enhancing PD-1 therapies by targeting vaccine-induced immunosuppressive DC.

## Introduction

Immune checkpoint inhibitors (ICI)-based immunotherapy has resulted in important advances, with some patients achieving long-term responses, which depend on the type of tumor and drugs used (1). Among others, 20% and 52% long-term survival has been reported for melanoma patients treated with ipilimumab (2) or ipilimumab plus nivolumab (3), respectively, 40% for non–small-cell lung cancer patients treated with ipilimumab plus nivolumab (4) and 52% for renal cell carcinoma patients treated with ipilimumab plus nivolumab (5). However, new alternative strategies are needed for non-responder patients. Since patients with a poor tumor lymphocytic infiltrate have a low response rate (6, 7), strategies are being developed to inflame cold tumors and increase their responsiveness to ICI. In addition to tumor immunogenic cell death promoted by chemotherapy and radiotherapy, vaccination may also specifically activate anti-tumor immunity (8). Vaccination was used in cancer patients (9), but it has provided a limited therapeutic effect. The tumor immunosuppressive environment and vaccine-associated immunomodulatory mechanisms restrain lymphocyte priming, resulting in poorer vaccine immunogenicity. We have previously reported the induction of a subset of immunoregulatory dendritic cells (DC) during vaccination in murine models and in DC-based immunization clinical protocols (10, 11). These DC, denominated DC-IL10^+^ (10), have poor T-cell stimulatory capacity, associated with lower expression of MHC and costimulatory molecules, and upregulation of immunomodulatory factors like IL-10, PD-L1 and Tyro/Axl/Mer (TAM) receptors. Since blockade of these immunosuppressive factors improved vaccine efficacy, our aim was to systematically analyze gene expression in DC-IL-10^+^, in order to identify other druggable targets, modulate them and enhance vaccine efficacy. Although we have identified molecules with therapeutic potential, most interesting findings came from the characterization of DC-IL-10^+^ origin, involving a monocytic precursor. Interestingly, monocyte depletion with gemcitabine (12), inhibited DC-IL-10^+^ generation, as an alternative to blocking molecules involved in DC-IL-10^+^ suppressive actions. Therefore, pre-vaccination treatment with gemcitabine, which reduced DC-IL-10^+^generation, led to a stronger priming of antitumor immunity, and concomitant superior therapeutic efficacy in tumor bearing mice. Moreover, this optimized vaccination protocol improved therapeutic responses induced by anti-PD-1 antibodies, with variable results depending on tumor type, suggesting the efficacy of this vaccine and the necessity of personalizing these combinations to optimize their efficacy.

## Materials and methods

### Reagents

OVA (98% purity, endotoxin-free; Hyglos, Germany) was used with adjuvants Imiquimod (Meda-Aldara™) or poly(I:C) (Amersham). Peptides OVA(257-264), OVA(323-339), AH1-A5 and ELA (>90% purity) were from Genecust (Boynes, France). Gemcitabine (Pfizer SL; Madrid, Spain), clodronate and control liposomes (Liposoma^®^) and anti-Ly6C (clone Monts1; BioXcell) were used for depletion experiments.

### Mice

Eight weeks-old female IL-10 reporter Vert-X (B6(Cg)-Il10tm1.1Karp/J) mice (Jackson), and C57BL/6J and BALB/c mice (Envigo; Barcelona, Spain) were used. HHD-DR1 mice (B2m^tm1Unc^ H2-Ab1^tm1Doi^ Tg(HLA-A/H2-D/B2M)1Bpe Tg(HLA-DR1)/Orl) were obtained from Dr. F. Lemonnier (Paris, France) and bred in our facilities. They were maintained in pathogen-free conditions and treated according to guidelines of the institution, after study approval by the review committee (protocol 101-19).

### Cell lines

Lewis lung adenocarcinoma cells expressing OVA (LLC-OVA) were obtained after stable transduction of LLC cells (ATCC) with a lentiviral vector encoding OVA (13) (gift from D. Escors; Navarrabiomed-Biomedical Research Center, Pamplona, Spain). B16-OVA cells were from Dr. G. Kroemer (Paris, France). Cells were grown in complete medium RPMI-1640 (Lonza^®^) plus 10% (v/v) fetal bovine serum and penicillin/streptomycin. Re-authentication of cells was not performed since receipt. Cells were periodically tested for mycoplasma contamination.

### Immunization

Mice were subcutaneously (s.c.) injected with OVA (0.5 mg/mouse; day 0) or peptides AH1-A5 or ELA (20-100 μg; days 0–2) combined with adjuvants: topical Imiquimod cream (2.5 mg/mouse; days 0–2) or poly(I:C) (50 μg/mouse; s.c.; day 0). Some mice received intraperitoneal (i.p.) injection of gemcitabine (30 mg/kg; day -1 and 0), clodronate or control liposomes (i.v.; 10 ml/kg; day -1) or anti-Ly6C (i.p.; 5 mg/kg; day -1). Mice were sacrificed at day 2 after immunization when testing DC depletion or at day 7 for immunogenicity assays.

### Tumor treatment experiments

Mice were s.c. injected with 2 x 10^6^ LLC-OVA or 5 x 10^5^ B16-OVA cells. Six days later (when tumors were around 4-6 mm in diameter), they received 2 cycles of OVA (intratumor; 0.5 mg/mouse; day 0) plus Imiquimod at day 0–2 as described above, with or without gemcitabine (30 mg/kg; day -1 and 0). Some groups received 100 μg/mouse of anti-PD-1 (clone RMP1-14) or its corresponding IgG2a isotype control (clone 2A3) (all from BioXcell) every 3 days. Tumor volume was calculated as V= (length x width^2^)/2. Mice were euthanized when tumors reached 16 mm in diameter in survival assays, or 7 days after immunization for immune response analyses.

### IFN-γ ELISPOT

Responses induced by vaccination were measured using IFN-γ ELISPOT Set (BD-Biosciences). Splenocytes (5 × 10^5^/well) were stimulated with OVA(257-264), OVA (323-339), AH1-A5 or ELA (10 μM) for 24 hours at 37 °C and the number of spot-forming cells was counted using an ImmunoSpot automated counter. Non-stimulated cells were used as controls.

### *In vitro* assays to assess the effect of gemcitabine on DC

Bone marrow-derived DC (BMDC) were differentiated from precursors as described (11). Day 6 BMDC were treated with Imiquimod (*In vivo*gen) (10 μg/ml) and gemcitabine (2.5 μM), and supernatants were harvested 24 hours later. IL-10 content was measured by OptEIA™ Set (BD Biosciences) following manufacturer’s instructions.

### Flow cytometry

Spleens and tumors were homogenized and cells were incubated for 10 min with Fc Block™ (BD-Biosciences) and stained with antibodies (**Supplementary Table S1**). Lymphocyte functions were analyzed after 4 hour stimulation with OVA(257-264) or OVA(323-339) (10 μM) in the presence of GolgiStop and GolgiPlug (BD-Biosciences). For cytoplasmic and intranuclear staining, BD Cytofix/Cytoperm™ kit and Foxp3/Transcription Factor Staining Buffer Set (eBioscience™) were used. Non-stimulated cells were used as controls. Samples were acquired with Cytoflex cytometer (Beckman Coulter) and data were analyzed using FlowJo software (Tree Star Inc.).

### DC isolation

Spleens of Vert-X mice were processed using Collagenase/DNase and CD11c^+^ cells were isolated using MagniSort™ Mouse CD11c Positive Selection Kit and autoMACS^®^ Pro Separator. Next, cells were stained and IL-10^+^ and IL-10^-^ CD11c^+^, I-A^b+^ cells were sorted using FACSAria flow cytometer, after excluding CD3^+^, NKp46^+^ and CD19^+^ cells. Purified DC were lysed and stored in RLT buffer (Qiagen).

### RNAseq

RNAseq experiments were carried out using purified DC from Vert-X mice and tumor extracts from C57BL/6J mice treated according to different protocols (Supplementary Methods).

### Gene expression analyses

RNAseq data analysis was performed as described (14), with minor modifications. Sample quality was verified using FastQC; alignment of reads to the mouse genome (mm10) was performed using STAR (15); gene expression quantification using read counts of exonic gene regions was carried out with featureCounts; the gene annotation reference was Gencode; and differential expression statistical analysis was performed using R/Bioconductor (16). Gene expression data were normalized with edgeR and voom. After quality assessment and outlier detection with R/Bioconductor, a filtering process was performed. Genes with <6 read counts in more than 50% of the samples were considered as not expressed. LIMMA (Linear Models for Microarray Data) was used to identify genes with significant differential expression between conditions (Fold-change>2 and adjusted P <0.05). Data can be accessed at GEO (GSE198973, GSE198974 respectively). Gene ontology analysis and Gene set enrichment analysis (GSEA) were performed using Panther (http://www.pantherdb.org) and www.gsea-msigdb.org. Enrichment analyses were carried out using the Molecular Signatures Database (MSigDB) as well as signatures extracted from Immgen (https://www.immgen.org).

### Statistical analysis

Statistical analysis was performed using GraphPad Prism (GraphPad) v7. After checking for normality, T-tests and ANOVA, or non-parametric tests, were used. LogRank tests were used for survival analysis. A P < 0.05 was taken to represent statistical significance.

## Results

### IL-10-producing DC display a distinct gene expression pattern

**Figure 1:**
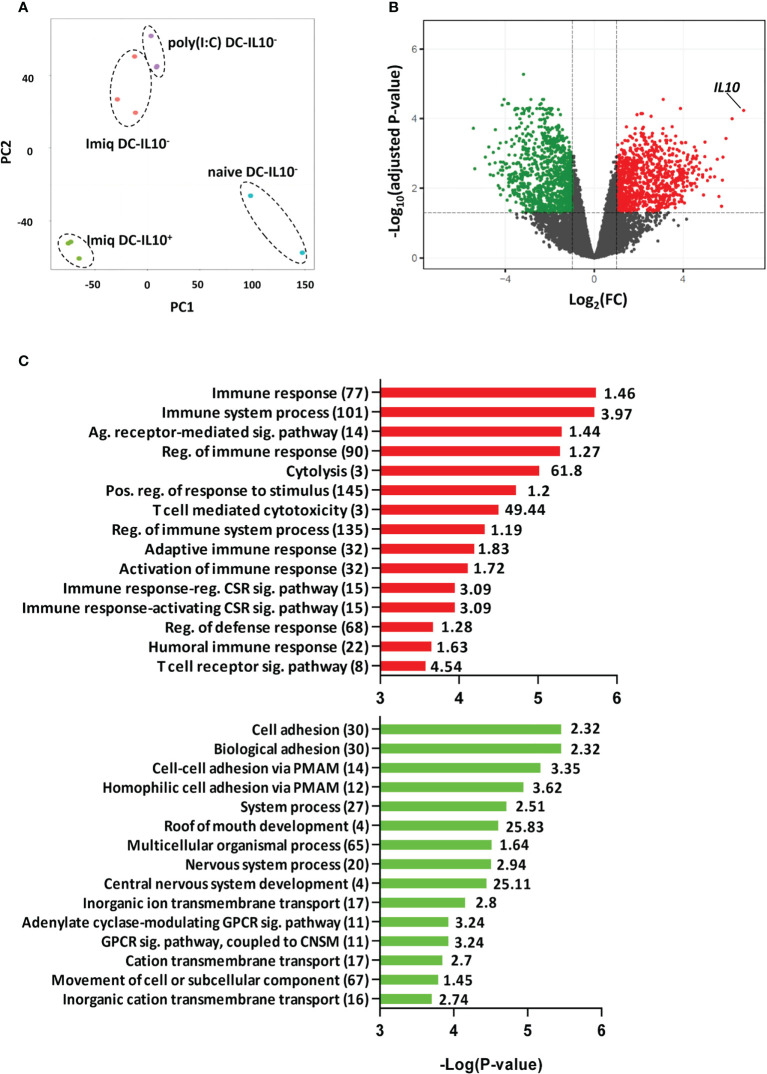
DC IL-10^+^ have a different transcriptomic profile. Splenic DC from Vert-X mice (naïve or immunized with OVA + Imiquimod or poly(I:C) adjuvants) pooled from 10-20 mice/group, were purified according to their IL-10 production profile and subjected to RNAseq experiments. **(A)** Principal component analysis (PCA) of the different treatment DC groups. **(B)** Volcano plot showing DEGs between DC IL-10^+^ and DC IL-10^-^ obtained from Imiquimod vaccinated mice. Colored dots represent significant (FC>2, adjusted P <0.05) DEGs between groups, with green dots representing down-regulated genes and red dots up-regulated genes in DC IL-10^+^. **(C)** Most significantly enriched GO terms in up-regulated and down-regulated genes according to Panther. Values in brackets indicate the number of genes classified under a particular GO term, with the enrichment value shown to the right. (Ag, Antigen; Sig, Signaling; Reg, Regulation; Pos, Positive; CSR, Cell Surface Receptor; PMAM, Plasma Membrane Adhesion Molecule; GPCR, G protein-coupled receptor; CNSM, Cyclic nucleotide second messenger).

To identify potential targets in DC-IL-10^+^, we first carried out RNAseq experiments with DC obtained from IL-10 reporter Vert-X mice. We used DC from naive mice or from mice immunized with OVA+Imiquimod or OVA+poly(I:C). DC-IL-10^+^ were only generated in Imiquimod-immunized mice and principal component analysis showed that they clustered apart from the other DC subsets. DC-IL-10^-^ from Imiquimod or poly(I:C)-immunized mice grouped together, but separated from DC from naive mice (**Figure 1A**). Therefore, for subsequent analyses we compared DC-IL-10^+^ and DC-IL-10^-^ from Imiquimod-immunized mice. This comparison revealed 923 genes upregulated in DC-IL-10^+^ (with IL-10 in a prominent position) and 1052 genes upregulated in DC-IL-10^-^ (**Figure 1B**). GO analyses of genes upregulated in DC-IL-10^+^ included terms associated with Immune response, Immune system process, Adaptive immune response and Activation and Regulation of immune response, in agreement with their capacity to regulate immune processes (**Figure 1C**, top). However, terms associated with DC-IL-10^-^ were mainly related to Cell adhesion (**Figure 1C**, bottom), possibly related to their role as stimulatory antigen presenting cells.

### Single molecule targeting in DC-IL-10^+^ is not effective at improving immune response

Blockade of suppressive molecules overexpressed by DC-IL-10^+^ improves vaccine immunogenicity (10, 11). To identify additional targetable molecules in DC-IL-10^+^, we selected genes upregulated in DC-IL-10^+^ that could be potentially involved in their immunosuppressive capacity and had available inhibitors. This included cytokine/cytokine receptors, enzymes, transcription factors and inflammatory mediators (**Supplementary Figure S1A**). As opposed to the results obtained blocking IL-10, PD-1/PD-L1 or TAM receptors, immunization with OVA+Imiquimod in the presence of single inhibitors against these targets did not improve vaccine immunogenicity (**Supplementary Figure S1B**). Of note, Rhein, a MSR1 inhibitor (17), significantly decreased responses as compared to control.

### Most DC-IL-10^+^ have a monocytic origin

**Figure 2:**
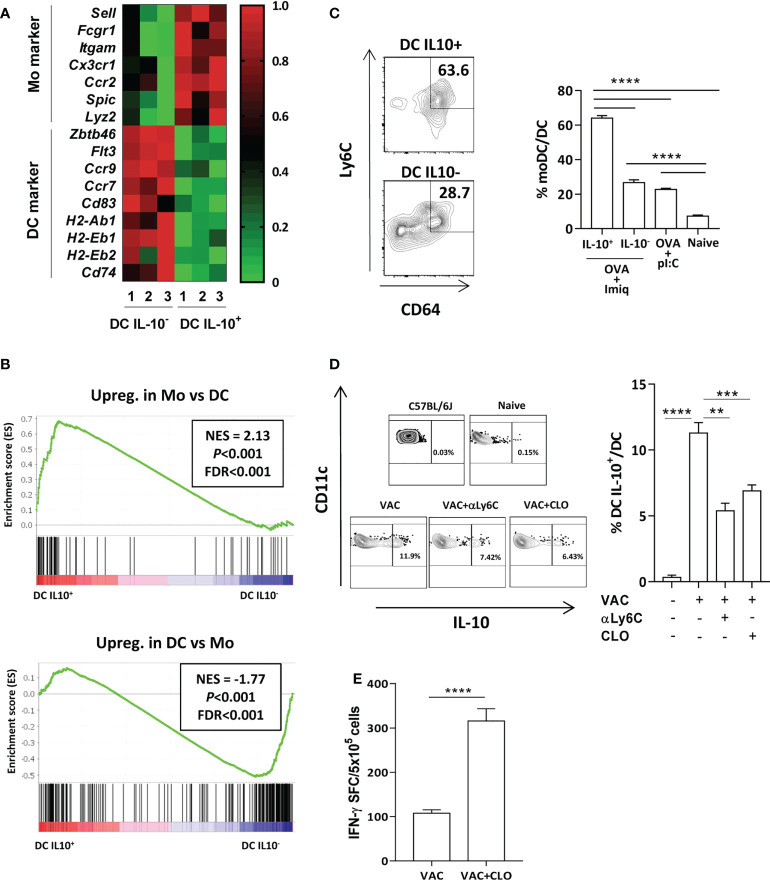
Most DC IL-10^+^ have a monocytic origin. **(A)** Expression heatmap of monocyte- and DC-associated genes in DC IL-10^+^ vs DC IL-10^-^ according to RNAseq data. **(B)** Gene set enrichment analysis of genes found in DC IL-10^+^ using gene signatures from the Immgen database observed in monocytes (upper panel) or in DC (lower panel) NES: normalized enrichment score. Statistical parameters (P and FDR) were provided by the GSEA webpage, calculated as described in Subramanian et al. (19). **(C)** Expression of monocyte markers in splenic DC from vaccinated Vert-X mice according to their IL-10-production capacity. Representative plots (left) and summary (4-8 mice/group). **(D)** Vert-X mice were immunized with OVA + Imiquimod (VAC) with or without monocyte-depleting agents: clodronate (CLO) or anti-Ly6C antibodies (αLy6C). DC were gated as CD11c^high^, I-A^b high^ cells and the percentage of DC IL-10^+^ was calculated for each group. A control untreated C57BL/6J mice is also shown. Representative plots (left panels) and summary (n=4 mice/group). **(E)** C57BL/6J mice (n=6-8/group) were immunized with OVA + Imiquimod with or without clodronate and one week later responses against OVA(257-264) CD8 T-cell epitope were determined by ELISPOT, measuring the number of spot forming cells (SFC). Bars show mean + SEM (**, P < 0.01; ***, P < 0.001; ****, P < 0.0001).

We previously showed that DC with upregulated TAM receptor expression, a feature of DC-IL-10^+^, were monocyte derived (11). Moreover, immunosuppressive DC identified in a viral infection model had a monocytic origin (18). Interestingly, RNAseq data indicated that DC-IL-10^+^ also derived from monocytes. Selectins (*Sell*), integrins (*Itgam*), chemokine receptors (*Ccr2*, *Cx3cr1*) and other monocytic markers (*Spic*, *Lyz2*) were upregulated in DC-IL-10^+^ (**Figure 2A**). However, DC-associated markers *Zbtb46*, *Flt3*, chemokine receptors *Ccr7* and *Ccr9*, MHC class II molecules (*H2-Ab1, H2-Eb1* and *H2-Eb2*), Invariant chain *Cd74* and the DC maturation marker *Cd83*, were upregulated in DC-IL-10^-^. Moreover, more comprehensive gen set enrichment analyses using gene datasets of differentially expressed genes (DEGs) between DC and monocytes obtained from Immgen (**Supplementary Table S2**) corroborated the monocytic origin of DC-IL-10^+^ (**Figure 2B**). In addition, we compared the gene expression profile of our DC with data from immunosuppressive DC generated in a chronic viral infection (18). Cells induced in this context, as opposed to stimulatory DC, have a monocytic origin, and are characterized by IL-10 expression, upregulation of PD-L1 and poor stimulatory capacity. These analyses demonstrated a significant gene set enrichment, supporting again the monocytic origin of DC-IL-10^+^ (**Supplementary Figure S2**). These results were confirmed *via* flow cytometry. More than 60% of DC-IL-10^+^ generated after OVA + Imiquimod vaccination derived from monocytes, while only around 25% in the case of DC-IL-10^-^, and DC-IL-10^-^ from OVA+poly(I:C)-vaccinated mice (**Figure 2C**) had this origin. In order to distinguish DC-IL-10^+^ from macrophages, we measured F4/80 expression and observed that, while an important percentage of F4/80^+^ macrophages belong to the CD11b^+^ subset, DC-IL-10^+^ expressed very low levels of F4/80 (**Supplementary Figure S3**).

Finally, we used as monocyte depleting agents clodronate liposomes or anti-Ly6C before vaccination. Although under these experimental conditions they induced a reduction in total CD11b^+^ myeloid cells, this mainly corresponded to monocytes, since decrease of macrophage proportions did not reach statistical significance (**Supplementary Figure S4**, upper panels). According to this monocyte depletion, lower proportions of monocyte-derived DC were observed, mainly in mice treated with clodronate (**Supplementary Figure S4**, lower panels). In agreement with a potential monocytic origin of DC-IL-10^+^, we observed that mice treated with clodronate or antiLy6C had lower proportions of DC-IL-10^+^ (**Figure 2D**). Moreover, pretreatment with clodronate resulted in a higher vaccine immunogenicity (**Figure 2E**). Anti-Ly6C was not included in these experiments, since TCR stimulation upregulates Ly6C on T cells (20). Thus, in the absence of monocytes, the generation of DC-IL-10^+^ is reduced, facilitating induction of a more potent immunity.

### Gemcitabine depletes DC-IL-10^+^ and improves vaccine potency

**Figure 3:**
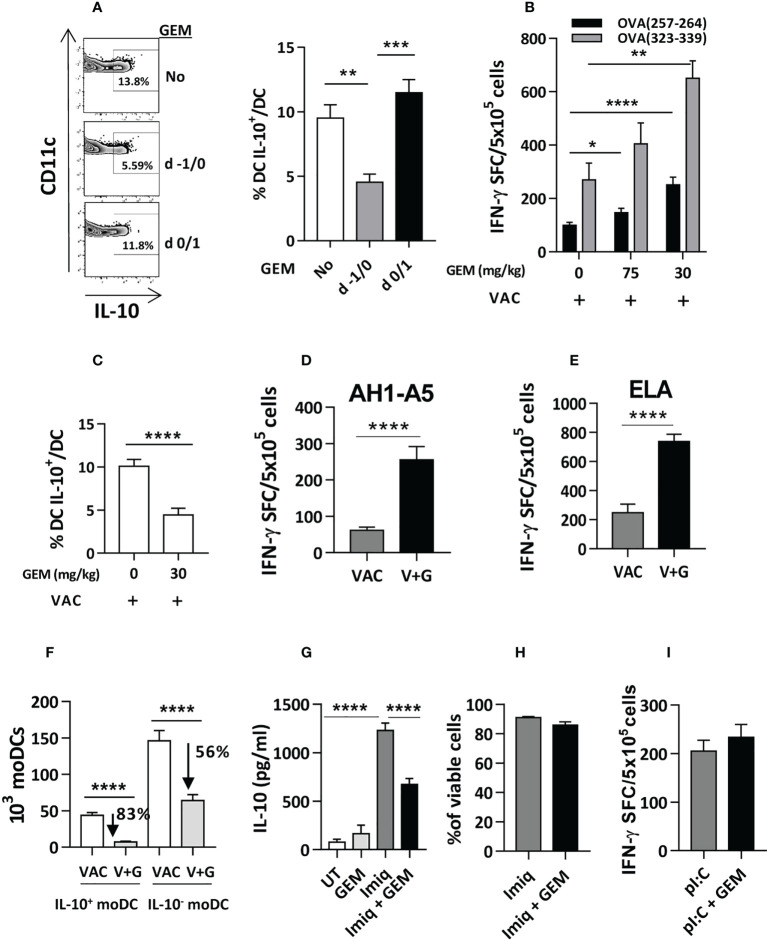
Gemcitabine administration reduces generation of DC IL-10^+^ and improves vaccine immunogenicity. **(A)** Gemcitabine (GEM) was administered to Vert-X mice (n=4-8/group) before (days -1/0) or during (days 0/1) vaccination with OVA and two days later the percentage of splenic DC IL-10^+^ was measured. **(B)** Different doses of gemcitabine were administered to C57BL/6J mice (n=4-12/group) before vaccination with OVA (VAC) and responses against OVA(257-264) and OVA(323-339) peptides were determined by an IFN-gamma ELISPOT, measuring the number of spot forming cells (SFC). **(C)** Effect of administration of 30 mg/kg of gemcitabine on DC IL-10^+^ generation before vaccination (VAC) of C57BL/6J mice (n=12/group). **(D)** BALB/c or **(E)** HHD mice (n=4-7 mice/group) were vaccinated with peptide AH1-A5 or ELA, respectively (VAC) or received the vaccine plus gemcitabine (V+G). One week later responses against the immunizing peptides were determined by ELISPOT. **(F)** Effect of gemcitabine administration on the generation of DC IL-10^+^ and DC IL-10^-^ (n=12 mice/group) according to their monocytic origin. Bone marrow-derived DC were left untreated (UT), treated with Imiquimod, gemcitabine or both, and IL-10 secretion (n=12 wells/condition) **(G)** and cell viability (n=4) **(H)** were determined. **(I)** C57BL/6J mice (n=8-10/group) were immunized with OVA + poly(I:C) with or without gemcitabine and responses against OVA(257-264) peptide were determined by ELISPOT. Bars show mean + SEM (*, P < 0.05; **, P < 0.01; ***, P < 0.001; ****, P < 0.0001).

Gemcitabine, a chemotherapeutic agent approved for different tumors (12), has immunomodulatory properties (21, 22), including depletion of some myeloid cells (23). Thus, gemcitabine administration at a dose of 75 mg/kg at days -1 and 0 pre-vaccination significantly inhibited the DC-IL-10^+^ generation, whereas administration at days 0 and 1 had no effect (**Figure 3A**), indicating the necessity of a previous monocyte depletion to prevent DC-IL-10^+^ generation. Depletion resulted in enhanced vaccine-induced CD8 and CD4 T-cell responses, even when using a lower dose of 30 mg/kg (**Figure 3B**), which still impaired generation of DC-IL-10^+^ (**Figure 3C**). Equivalent enhanced vaccine immunogenicity was obtained using peptides AH1-A5 (target in breast (24) and colon cancer (25) models in BALB/c mice) (**Figure 3D**), and ELA [from Melan-1/MART antigen and used in human melanoma vaccination experiments (26)], tested in HHD-DR1 mice (**Figure 3E**).

Analysis of the effect of gemcitabine on IL-10^+^ and IL-10^-^ moDC subsets, revealed that gemcitabine reduced splenic IL-10^+^ moDC numbers by 83%, as opposed to a 56% reduction of IL-10^-^ moDC (**Figure 3F**). *In vitro* experiments with gemcitabine and Imiquimod showed that gemcitabine reduced Imiquimod-induced IL-10 production (**Figure 3G**), unrelated to gemcitabine toxicity on DC (**Figure 3H**). Finally, gemcitabine administration previous to immunization with poly(I:C) as adjuvant (where all moDC are DC-IL-10^-^), did not improve vaccine immunogenicity (**Figure 3I**), suggesting that its effect occurs mainly through DC-IL-10^+^. These results indicate that gemcitabine impairs the generation of IL-10-producing DC, which could be partially explained by an effect on IL-10 production, resulting in stronger T-cell responses.

### Gemcitabine improves the therapeutic effect of vaccination associated with enhanced T-cell immunity

**Figure 4:**
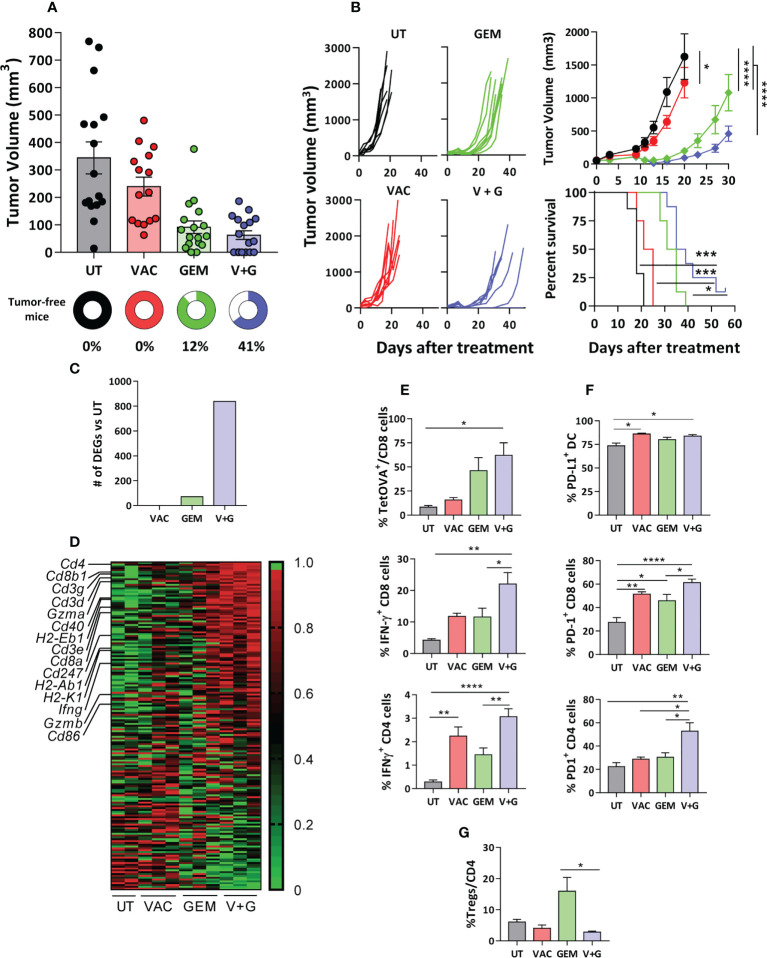
Gemcitabine improves the therapeutic effect of vaccination associated with enhanced T-cell immunity. C57BL/6J mice bearing 5-6 mm LLC-OVA tumors were untreated (UT) or treated with OVA + Imiquimod vaccine (VAC), gemcitabine (GEM) or the combination (V+G). **(A)** Tumor volume and the percentage of tumor-free mice were determined at day 11 of treatment (n=15-17 mice/group). **(B)** Tumor growth curves and survival were studied in long-term experiments (n=8 mice/group). **(C)** Representative tumors (n=3/group; except UT, n=2) from mice treated for one week were obtained and gene expression was analyzed by RNAseq. Bars correspond to differentially expressed genes with respect UT mice (FC>2, adjusted P <0.05). **(D)** Gene expression heatmap corresponding to genes included in the GSEA hallmark dataset “Allograft rejection” in the four groups. Tumors (n=5-8/group) were obtained after one week of treatment and % of TetOVA^+^ cells and IFN-γ-producing CD8 and CD4 T-cells **(E)**, PD-L1^+^ DC and PD-1^+^ CD8 and CD4 cells **(F)** and Tregs **(G)** were determined by flow cytometry. Bars show mean + SEM (*, P < 0.05; **, P < 0.01; ***, P < 0.001; ****, P < 0.0001).

We next tested the therapeutic effect of the gemcitabine-containing vaccine in mice bearing relevant tumors. We discarded the 4T1 breast cancer model, since depletion of its high number of myeloid derived suppressor cells (MDSC) could mask its effect on vaccine-induced DC. The LLC lung cancer model is resistant to ICI immunotherapy (27) and has lower MDSC levels (10% of splenocytes) than 4T1 or CT26 models (30-40%) (28). We generated an OVA-expressing LLC tumor, specifically recognized by OVA-immunized mice (**Supplementary Figure S5A**) and with 5% splenic MDSC (**Supplementary Figure S5B**). Short-term therapeutic vaccination showed that, whereas vaccination alone barely affected tumor growth, gemcitabine decreased tumor size. The vaccine and gemcitabine combination rejected a higher number of tumors (7 out of 17 (41%) in the combination vs 2 out of 17 (12%) in the gemcitabine group) (**Figure 4A**). Long-term survival experiments showed that untreated mice and mice treated with monotherapies died before day 40, while 25% of mice survived in the combination treatment group (**Figure 4B**).

We next investigated the possible factors associated with the enhanced efficacy of the combination treatment compared to the vaccine and gemcitabine monotherapies. By using samples obtained from tumor-bearing Vert-X mice after a single treatment cycle, we confirmed that addition of gemcitabine to vaccination decreased the number of DC-IL-10^+^, not only in the spleen but also in tumor-draining lymph nodes (**Supplementary Figure S6**). RNAseq studies of tumor samples of C57BL/6J mice after a single treatment cycle showed that the combination group had the highest number of DEGs when compared with untreated mice (**Figure 4C**). GSEA revealed that the Allograft Rejection signature from the GSEA Hallmark was common to all comparisons (**Supplementary Figure S7**). Thus, genes related to T-cell subsets (*Cd4*, *Cd8a*, *Cd8b1*), CD3 and ζ chain (*Cd3g*, *Cd3d*, *Cd3e*, *Cd247*), T-cell effector molecules (*Ifng*, *Gzma*, *Gzmb*) and mature DC (*H2-Eb1*, *H2-Ab1*, *H2-K1*, *Cd40, Cd86*) were strongly upregulated in the combination group (**Figure 4D**). Flow cytometry confirmed that the combination group had the highest levels of OVA(257-264)-specific CD8 T-cells (TetOVA), as well as IFN-γ-producing OVA-specific CD8 and CD4 T-cells (**Figure 4E**). Similar findings were observed in splenic cells, with increased proportion of OVA-specific cytokine-producing CD8 and CD4 T-cells in the combination group, but not in Tregs (**Supplementary Figure S8**). Interestingly, the combination group had the highest percentages of PD-1^+^ tumor CD4 and CD8 T cells (**Figure 4F**). Notably, although gemcitabine enhanced the number of infiltrating Tregs, its combination with vaccination did not show such increase in Treg percentages (**Figure 4G**).

Regarding myeloid cells, no significant changes were observed in the percentages of tumor CD11b^+^ cells or total DC (**Supplementary Figures S9A, B**). In the case of DC IL-10^+^, studies carried out in Vert-X mice with LLC-OVA tumors showed that, as in the spleen or draining lymph nodes, higher levels were observed after vaccination, but they decreased in the combination group, although this did not reach statistical significance (P=0.1) (**Supplementary Figure S9C**). Furthermore, more than 80% of tumor DC were PD-L1^+^ in most groups (**Figure 4F**). These results provided a suitable scenario for the addition of PD-1 blocking antibodies to the combination.

### The vaccine + gemcitabine combination enhances the therapeutic efficacy of PD-1 blockade in a tumor-dependent manner

**Figure 5:**
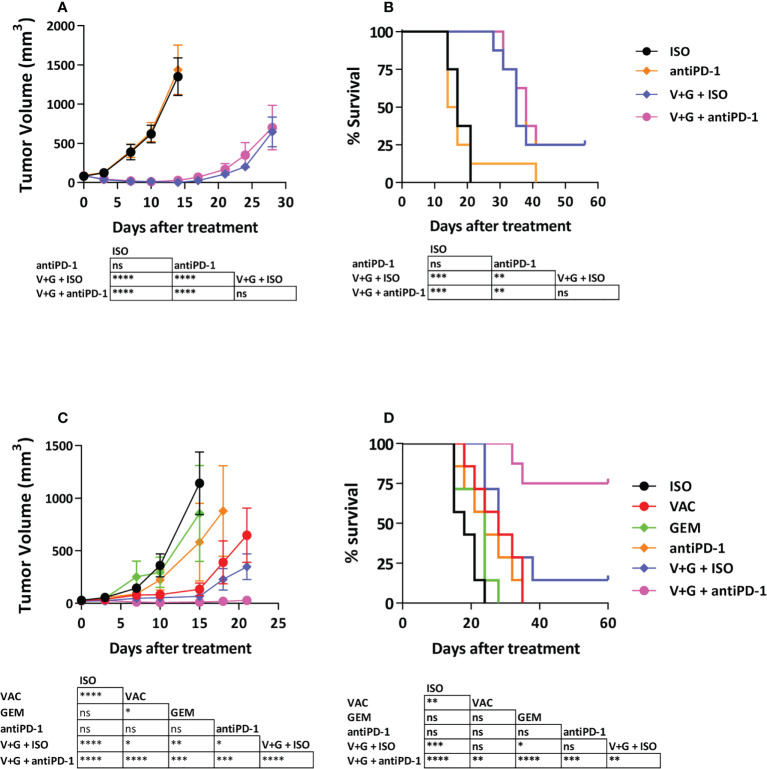
Antitumor efficacy of the combination Vaccine + Gemcitabine with PD-1-blocking antibodies. C57BL/6J mice (n=7-8/group) with 5-6 mm LLC-OVA **(A, B)** or B16-OVA **(C, D)** tumors were treated with different combinations of vaccine, gemcitabine, anti-PD-1 antibodies or isotype control antibodies. Tumor growth **(A, C)** and animal survival **(B, D)** were evaluated twice per week. (*P < 0.05; **P < 0.01; ***P < 0.001; ****P < 0.0001). ns, non significant.

We tested if combination treatment with vaccine + gemcitabine could improve responses to anti-PD-1 therapy in LLC-OVA and B16-OVA tumors, known to be poor responders to anti-PD-1. As reported for parental LLC tumors (27), anti-PD-1 did not delay LLC-OVA tumor growth, whereas vaccine + gemcitabine significantly delayed tumor growth, increasing long-term survival (P<0.001). Surprisingly, although combination of vaccine + gemcitabine with anti-PD-1 was superior to anti-PD-1 monotherapy (P<0.01), it did not show improved efficacy over vaccine + gemcitabine (**Figures 5A, B**), even at higher anti-PD-1 doses (**Supplementary Figure S10**). In B16-OVA tumors, monotherapies with gemcitabine or anti-PD-1 had no significant anti-tumor effect, whereas vaccination, and to a greater extend, vaccination + gemcitabine (P<0.001), significantly delayed tumor growth. As opposed to LLC-OVA, the effect of this combination was more dependent on vaccination than on gemcitabine. Interestingly, the triple combination vaccination + gemcitabine and anti-PD-1 clearly reduced tumor growth (P<0.001 vs remaining groups) and prolonged survival (P<0.01 vs VAC+GEM; P<0.001 vs anti-PD-1), with 75% of mice alive and tumor-free at day 60 (**Figures 5C, D**). These data indicate that combining vaccination with gemcitabine and ICI can significantly delay B16-OVA tumor growth.

### CD8 T-cells in non-responder mice to combinations with anti-PD-1 display an exhausted phenotype with multiple inhibitory receptors

**Figure 6:**
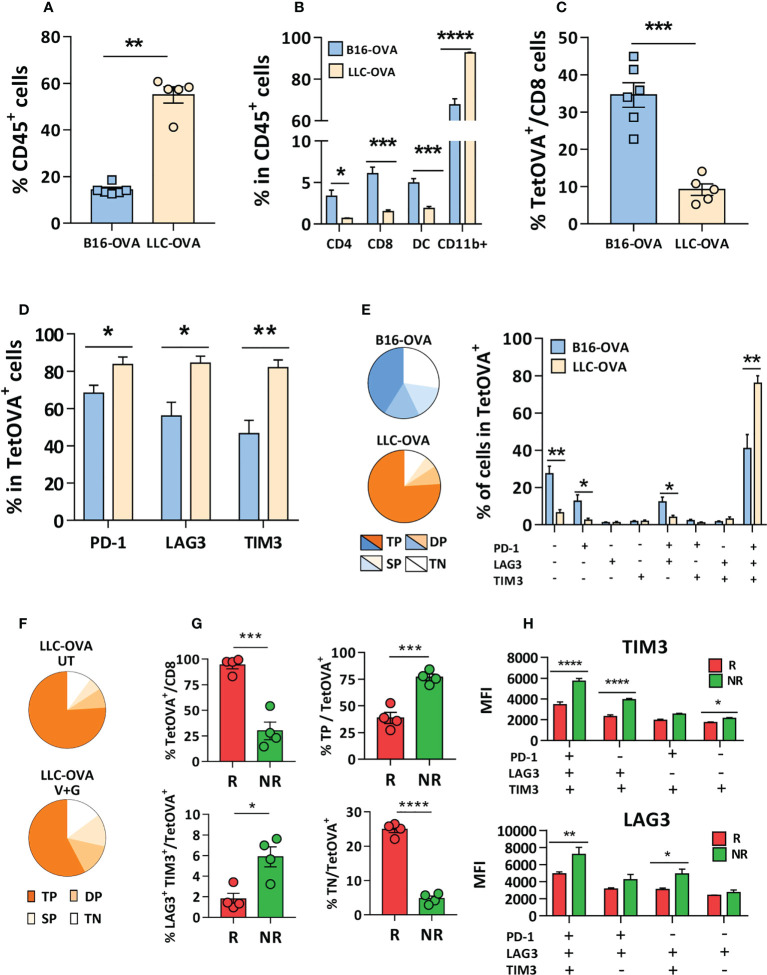
CD8 T-cells in non-responder mice to combinations with anti-PD-1 display an exhausted phenotype with multiple inhibitory receptors. Five mm LLC-OVA or B16-OVA tumors (n=5-6/group) were obtained from untreated C57BL/6J mice and flow cytometry was used to determine **(A)** the CD45^+^ leukocytic infiltrate, **(B)** the proportion of CD4, CD8, DC and myeloid CD11b^+^ cells and **(C)** TetOVA^+^ cells in CD8 cells. In the case of TetOVA^+^ cells, the proportion of cells expressing PD-1, LAG3 or TIM3 **(D)** and the combined expression of these markers is shown grouped as pie chart (triple negative, TN; single positive, SP; double positive, DP; triple positive, TP) or in individual bars **(E)**. Mice with LLC-OVA tumors (n=8/group) were treated for one week with vaccine + gemcitabine (V+G) or left untreated (UT) and the proportion of TN, SP, DP and TP TetOVA^+^ cells was determined by flow cytometry **(F)**. Percentage of TetOVA^+^ cells, TP, DP (LAG3^+^TIM3^+^) and TN cells in mice responding (R) or not (NR) (n=4/group) to vaccine + gemcitabine treatment after one week **(G)** and the fluorescence intensity **(H)** of LAG3 and TIM3 in TetOVA^+^ cells positive for these markers. Bars show mean + SEM (*, P<0.05; **, P < 0.01; ***, P < 0.001; ****, P < 0.0001).

To elucidate the different behavior of LLC-OVA and B16-OVA tumor-bearing mice we characterized several tumor features. *In vitro* experiments with tumor cells demonstrated a higher sensitivity of LLC-OVA to gemcitabine (IC_50 =_ 329 nM) when compared to B16-OVA (IC_50 =_ 1895 nM) (**Supplementary Figure S11**), explaining its stronger *in vivo* antitumor effect. Regarding immune parameters, LLC-OVA contained a more enriched CD45^+^ infiltrate (**Figure 6A**), with an abundant group of myeloid cells (**Figure 6B**), but the proportion of CD4, CD8 T-cells and DC (**Figure 6B**) and Tregs (**Supplementary Figure S12**) was higher in B16-OVA tumors. Moreover, B16-OVA tumors contained more antigen-specific TetOVA^+^ CD8 T-cells (**Figure 6C**). Of note, LLC-OVA tumors had more TetOVA^+^ cells expressing PD-1, LAG3 or TIM3 (**Figure 6D**), and a combined analysis revealed a higher number of exhausted T-cells, with simultaneous expression of all three molecules in about 80% of TetOVA^+^ cells. By contrast, TetOVA^+^ cells in B16-OVA tumors contained more triple negative cells, and to a minor extend, cells expressing only PD-1 or PD-1 and LAG3 (**Figure 6E**). These data indicate that tumor infiltrating antigen-specific T cells had a more exhausted phenotype in LLC-OVA tumors compared to B16-OVA.

Further analysis of lymphocytes in LLC-OVA tumors treated with vaccine + gemcitabine showed a decrease of triple positive cells (PD-1^+^LAG3^+^TIM3^+^) and increase of triple negative cells (PD-1^-^LAG3^-^TIM3^-^), presumably reflecting the generation of non-exhausted cells with antitumor capacity (**Figure 6F**). Moreover, separate analysis of TILs in mice responding (R; volume < 500 mm^3^) or not (NR) to vaccine + gemcitabine therapy revealed that R mice had higher percentage of infiltrating TetOVA^+^ T-cells than NR mice. R mice also had less triple positive and double positive (TIM3^+^/LAG3^+^) cells and more triple negative cells (**Figure 6G**). Nonetheless, expression of these markers (measured as fluorescence intensity) was higher in triple positive cells and in some double-positive cells (**Figure 6H**) in NR mice. Overall, these data suggest that besides PD-1, antigen-specific cells in mice not responding to vaccine + gemcitabine combination have additional inhibitory receptors that would impede response to anti-PD-1 therapy.

## Discussion

Modulation of immunosuppressive mechanisms may enhance the efficacy of immunotherapy. Therefore, to enhance vaccine immunogenicity and further improve responses to ICI, we carried out a systematic gene expression analysis in DC-IL-10^+^, immunosuppressive DC induced during vaccination. RNAseq revealed a different expression profile for DC-IL-10^+^, identifying potential targets like cytokines (IL-27, TGF-β), enzymes (COX-1, CD39), receptors (PDGFR, PPAR-γ), etc, with known immunomodulatory properties. However, none of the inhibitors tested improved vaccine immunogenicity. Processing of samples for DC purification may induce non-specific DC activation or loss of DC surface markers, masking detection of relevant targets. This could be a potential explanation for our failure in identifying molecules/pathways whose inhibition would increase vaccine immunogenicity. Alternatively, although we did not confirm expression of differentially expressed genes at the protein level, we may hypothesize that a hierarchy of immune elements, as recently proposed for innate immunity in cancer (29) (in our case with immunosuppressive properties, like IL-10, PD-L1 or MERTK) emerges with a higher relevance than those included in the present studies.

Interestingly, expression analyses revealed that DC-IL-10^+^ have monocytic origin. Pro-immunogenic moDC may be generated in infections (30) or during inflammation (31), but under other circumstances (11, 18, 32) these moDC can adopt an immunosuppressive profile. Considering this, we demonstrated that monocyte depletion using gemcitabine, a chemotherapeutic drug, reduces the generation of DC-IL-10^+^ and enhances vaccine immunogenicity. Several effects have been described for gemcitabine, including direct antitumor effects (12), immunomodulatory (21) and MDSC-depleting capacity (33). Here, we demonstrate that it also eliminates monocytic precursors that originate immunosuppressive DC. Although we cannot discard the DC-IL-10^+^-independent effects on the efficacy of gemcitabine in our combination protocols, the enhanced immunogenicity in tumor-free mice and the use of tumor models with poor presence of MDSC suggest a relevant mechanism through inhibition of moDC generation. The depleting action of gemcitabine operates through DC-IL-10^+^, since no effects on vaccine immunogenicity were observed when using a poly(I:C)-containing vaccine, and possibly also associated with a lower IL-10 production, as shown *in vitro*. Data from preclinical models and patients (34, 35) show reduced IL-10 production associated with gemcitabine, pointing out to the relevance of the elimination of this important immunosuppressive mechanism used by DC-IL-10^+^ (10). On the other side, the gemcitabine-induced depleting effects, including not only monocytes, but also neutrophils, have to be considered. Neutropenia is a common effect after gemcitabine administration (36), which may lead to dose reduction. However, we believe that the doses proposed and its time of administration (only during vaccination) may not have adverse effects beyond those currently observed in patients treated with gemcitabine. Alternative strategies for the future, centered on monocyte depletion, would be more specific. In this regard, it has been recently reported the development of a drug-conjugated anti-CD64 antibody for tumor treatment (37), that could be also useful in our vaccination context.

The lack of IL-10 induction capacity by poly(I:C)-containing vaccine would justify the use of this adjuvant, which would not require IL-10 blockade or gemcitabine use, as we propose in our Imiquimod-based model. However, in addition to Imiquimod, there are additional vaccine adjuvants targeting other TLR molecules, which are in development in preclinical and clinical phases (e.g.TLR4 or TLR9 ligands), with known capacity to induce IL-10 (10, 38–40). With these adjuvants, the presence of IL-10 should be considered, and the use of strategies targeting this pathway would potentially boost their immune potency.

In addition to inhibiting the stimulatory capacity of APC, IL-10 has beneficial effects by enhancing functional properties of effector CD8 T cells or by inhibiting immunopathology generated during inflammatory diseases. However, we believe that our past protocol of single time point inhibition of IL-10 or the current blockade of generation of DC-IL-10^+^ during vaccination, should mainly affect priming capacity of APC.

In line with our final goal of improving ICI-based therapies, we tested the combination of vaccine + gemcitabine in anti-PD-1 poor responder tumor models such as LLC-OVA and B16-OVA (27, 41). Enhanced antitumor effects were observed, although the underlying mechanisms were different. LLC-OVA tumor growth was inhibited more by gemcitabine, whereas B16-OVA tumors were more sensitive to vaccination. Differential tumor cell sensitivity and their immune microenvironment (e.g. higher levels of myeloid CD11b^+^ cells in LLC-OVA, which may hamper immune stimulating therapies (42, 43), or the higher lymphocytic infiltrate in B16-OVA, suggestive of a less hostile environment for lymphocyte infiltration) may account for these differences. Interestingly, the combined therapy induced the highest levels of PD-1^+^ infiltrating T-cells, indicating its suitability to accompany PD-1-blocking therapies.

Strategies combining vaccine + gemcitabine with anti-PD-1 were superior to anti-PD-1 monotherapy. However, whereas in LLC-OVA tumors this was due to the vaccine + gemcitabine combination, in B16-OVA tumors, a synergistic effect was observed, with 75% of long-term surviving mice. Some clues would explain these differences. First, LLC-OVA tumors contain more CD11b^+^ myeloid cells, but lower levels of subsets associated with better responses to ICI, such as T-cells (6, 44, 45) and DC (46–48). Although vaccination may increase the number of infiltrating T-cells, the higher basal T-cell levels observed in B16-OVA may indicate an environment more favorable for lymphocyte infiltration. Second, percentages of T cells expressing PD-1, LAG3 or TIM3 are significantly higher in LLC-OVA tumors, with higher per-cell expression, characteristic of cells with reduced expansion capacity after PD-1 blockade (49). Moreover, LLC-OVA have more triple positive cells, indicative of a more exhausted status that requires multiple checkpoint blockade for reinvigoration (50–52). Third, mice with LLC-OVA tumors responding to vaccine + gemcitabine therapy showed a downregulation of the expression of inhibitory receptors. However, although optimized vaccines may improve immune checkpoint profile of T-cells, there is still an important percentage of cells with expression of multiple receptors, requiring combined blockade to achieve functional rescue.

In summary, monocyte depletion before vaccination prevents the generation of immunosuppressive DC, resulting in improved vaccines with enhanced antitumor activity. This optimized vaccine increases response rates to anti-PD-1, but it depends on the nature and characteristics of the tumor infiltrate, which may require additional inhibitors to reach full efficacy.

## Data availability statement

The datasets presented in this study can be found in online repositories. The names of the repository/repositories and accession number(s) can be found below: https://www.ncbi.nlm.nih.gov/, GSE198973, https://www.ncbi.nlm.nih.gov/, GSE198974.

## Ethics statement

The animal study was reviewed and approved by Comité de Ética de Experimentación Animal-Universidad de Navarra.

## Author contributions

DR, DL and PS conceived and designed experiments. DR, MR, LS, BA, JE, EG, DAj, YS, EC, FN, SB, DAl performed the *in silico*, *in vitro* and *in vivo* studies. DR, DL, SHS, JJL and PS analyzed data. DL and PS supervised the project. DAj and PS, funding. All authors contributed to the article and approved the submitted version.

## Funding

This work was funded by grants from Instituto de Salud Carlos III, Fondo Europeo de Desarrollo Regional “Una manera de hacer Europa” (PI17/00249, PI20/00260 and PI20/00419), Fundación Científica de la Asociación Española Contra el Cáncer, Ministerio de Ciencia e Innovación (PID2019-108989RB-I00), the “Murchante contra el cáncer” initiative and Gobierno de Navarra (Proyectos Estrategicos AGATA, ref 0011-1411-2020-000011 and 0011-1411-2020-000010; and 51-2021).

## Acknowledgments

Authors thank Drs. Kroemer and Escors for their gift of B16-OVA cells and plasmids, respectively.

## Conflict of interest

The authors declare that the research was conducted in the absence of any commercial or financial relationships that could be construed as a potential conflict of interest.

## Publisher’s note

All claims expressed in this article are solely those of the authors and do not necessarily represent those of their affiliated organizations, or those of the publisher, the editors and the reviewers. Any product that may be evaluated in this article, or claim that may be made by its manufacturer, is not guaranteed or endorsed by the publisher.
