# Supplementary material for: Gemcitabine-mediated depletion of immunosuppressive dendritic cells enhances the efficacy of therapeutic vaccination
Source: Front Immunol. 2022 Oct 10;13:991311. doi: 10.3389/fimmu.2022.991311 (PMC9589451; doi:10.3389/fimmu.2022.991311)
Supplement: Supplementary file 1 [file DataSheet_1.docx]

Supplementary Material

# Supplementary Data

**Supplementary Methods:**

*RNAseq*

In experiments involving DC obtained from VertX mice, total RNA was extracted from purified DC samples using QIAGEN RNeasy Micro Kit. RNA quality was assessed on an Agilent’s 4200 TapeStation System and samples with an RNA Integrity Number (RINe) > 8 were selected. RNA concentration was determined using Qubit 3.0 Fluorometer (Life Technologies). Bulk RNAseq was performed following MARS-Seq protocol adapted for bulk RNAseq (Jaitin et al, 2014) with minor modifications. Poly-A RNA was selected with Dynabeads Oligo (dT) (Ambion) and reverse-transcribed with AffinityScript Multiple Temperature Reverse Transcriptase (Agilent) using poly-dT oligos carrying a 7 bp-index. Up to 8 samples with similar overall RNA content were pooled together and subjected to linear amplification by IVT using HiScribe T7 High Yield RNA Synthesis Kit (New England Biolabs). Next, aRNA was fragmented into 250-350 bp fragments with RNA Fragmentation Reagents (Ambion) and dephosphorylated with FastAP (Thermo) following manufacturer´s instructions. Partial Illumina adaptor sequences were ligated with T4 RNA Ligase 1 (New England Biolabs), followed by a second RT reaction. Full Illumina adaptor sequences were added during final library amplification with KAPA HiFi DNA Polymerase (Kapa Biosystems). Libraries were sequenced in an Illumina NextSeq 500 at a sequence depth of 10 million reads per sample.

For tumor samples, RNA sequencing was performed by adapting the technology of SCRB-Seq (Bagnoli JW et al., 2018). Briefly, poly-(A)+ RNA were purified using the Dynabeads mRNA DIRECT Purification Kit (ThermoFisher Scientific). poly-(A)+ RNA were annealed to a custom primer containing a poly-(T) tract, a Unique Molecule Identifier (UMI), and a sample barcode. Retrotranscription using Template-switching oligonucleotides (TSO) was then used to synthetize and amplify 3’UTR enriched cDNA, resulting in barcoded cDNA fragments. Library preparation was performed using the Nextera XT library preparation protocol which introduces i5-P5 and i7-P7 structure for massive parallel sequencing. Quality control was performed following pre-amplification RT and library preparation to ensure quality and length accuracy, as well as to equilibrate sample pooling. Libraries were then sequenced using a NextSeq2000 sequencer (Illumina). 5-10 million pair-end reads (100 bp; Rd1:28; Rd2:75) were sequenced for each sample and demultiplexed using Cutadapt. RNAseq was carried out at the Genomics Unit of the Center for Applied Medical Research (CIMA, Universidad de Navarra).

**References**

Jaitin DA, Kenigsberg E, Keren-Shaul H, et al. Massively parallel single-cell RNA-seq for marker-free decomposition of tissues into cell types. Science. 2014;343:776–9.

Bagnoli JW, Ziegenhain C, Janjic A, et al. Sensitive and powerful single-cell RNA sequencing using mcSCRB-seq. Nat Commun. 2018;9:2937

| **2. Supplementary Figures and Tables**  **Supplementary Table S1:** Antibodies used for flow cytometry. | | | | | | | | | | |
| --- | --- | --- | --- | --- | --- | --- | --- | --- | --- | --- |
| **Targeted Molecule** | **Fluorochrome** | | **Working Dilution** (1:X) | | **Clone** | | **Supplier** | | **Reference** | |
| CD11b | BUV661 | | 500 | | M1/70 | | BD Biosciences | | 565080 | |
| CD11c | BV510 | | 200 | | HL3 | | BD Biosciences | | 562949 | |
| CD11c | BV421 | | 200 | | N418 | | BioLegend | | 117343 | |
| CD19 | PE Cy7 | | 500 | | 6D5 | | BioLegend | | 115530 | |
| CD25 | PE Cy7 | | 400 | | PC61.5 | | TONBO | | 60-0251-U100 | |
| CD335 | PE | | 400 | | 29A1.4 | | BD Pharmingen | | 560757 | |
| CD3e | APC | | 400 | | 145-2C12 | | BD Pharmingen | | 100312 | |
| CD3e | BUV496 | | 50 | | 145-2C11 | | BD Biosciences | | 564661 | |
| CD4 | Alexa700 | | 1000 | | RM4-5 | | BioLegend | | 100536 | |
| CD45 | APC-Cy7 | | 200 | | 30-F11 | | BioLegend | | 103116 | |
| CD64 | PE | | 200 | | X54-5/7.1 | | BioLegend | | X54-5/7.1 | |
| CD8a | BV421 | | 500 | | 53-6.6 | | BioLegend | | 100753 | |
| CD107a | FITC | | 500 | | 1D4B | | BD Pharmingen | | 553793 | |
| FoxP3 | APC | | 400 | | 3G3 | | TONBO | | 20-5773 | |
| I-A^b^ | Percp Cy5.5 | | 1000 | | M5/114.15.2 | | BioLegend | | 107625 | |
| I-A^b^ | FITC | | 200 | | AF6-120.1 | | BD Pharmingen | | 553551 | |
| LAG3 | BV650 | | 100 | | C9B7W | | BioLegend | | 125227 | |
| Ly6C | Alexa700 | | 1000 | | HK1.4 | | BioLegend | | 128024 | |
| OVA tet | APC | | 100 | | - | | MBL | | TB-5001-2 | |
| PD1 | Percp Cy5.5 | | 1000 | | 29F.1A12 | | BioLegend | | 135208 | |
| PD-L1 | PE | | 1000 | | MIH5 | | BD Pharmingen | | 558091 | |
| TIM3 | BV785 | | 100 | | RMT3-23 | | BioLegend | | 119725 | |
| F4/80 | BV650 | | 500 | | BM8 | | BioLegend | | 123149 | |
| IFNg | PE | | 1000 | | XMG1.2 | | BD Biosciences | | 562020 | |
| TNF | PE Cy7 | | 1000 | | MP6-XT22 | | BD Pharmingen | | 557644 | |
| Maleimide | IR-885 | | 1000 | | - | | Promokine | | PK-PF840-0-05 | |
| **Supplementary Table S2** | | | |  | |  | |  | |  |
| **Dataset of genes differentially expressed between monocytes and dendritic cells** | | | | | | | | | |  |
| **obtained from Immgen (www.immgen.org)** | | | | | |  | |  | |  |
|  | |  | |  | |  | |  | |  |
| ***Upreg Mo_vs_DC*** | | | |  | |  | |  | |  |
| ARHGEF37 | | PDE7B | | GM26569 | | H4C3 | |  | |  |
| S1PR5 | | MSANTD3 | | GM45694 | | KIF21A | |  | |  |
| TPPP3 | | GM45153 | | FAM20A | | CDKL2 | |  | |  |
| APOA4 | | TNFSF13OS | | GM45472 | | GM11737 | |  | |  |
| CYP2AB1 | | A530013C23RIK | | GM11634 | | GM45345 | |  | |  |
| APOA1 | | ANKRD66 | | UNC5D | | GM43774 | |  | |  |
| FABP2 | | SPINK1 | | GM48066 | | GM26877 | |  | |  |
| SGMS2 | | CYP4F14 | | APBB1 | | HOXB4 | |  | |  |
| GALNT9 | | LCT | | MEP1B | | EDIL3 | |  | |  |
| CHIL3 | | CES2A | | GM4070 | | VMN1R179 | |  | |  |
| GAD1-PS | | MUC13 | | 1700023H06RIK | | MFSD9 | |  | |  |
| FZD4 | | STEAP3 | | IGHA | | MYRF | |  | |  |
| GM36161 | | GM13479 | | SLC6A19 | | GM38125 | |  | |  |
| GM20658 | | GM15902 | | COL4A1 | | GM20511 | |  | |  |
| CEBPB | | CLEC2H | | TTC12 | | CSMD3 | |  | |  |
| LILRA5 | | NYNRIN | | CKMT1 | | GM10785 | |  | |  |
| BMX | | CELSR3 | | ARHGAP23 | | GM37069 | |  | |  |
| TREM2 | | KRT20 | | AOX3 | | SCN1A | |  | |  |
| RBP2 | | GM49499 | | SIX4 | | ANK3 | |  | |  |
| APOC3 | | GPX3 | | GM22270 | | MYO15B | |  | |  |
| AI839979 | | GM42731 | | LRFN5 | | 3830403N18RIK | |  | |  |
| PRAP1 | | GM13814 | | ADARB1 | | SLC6A20B | |  | |  |
| LY6A2 | | GM14321 | | GM36756 | | H3C6 | |  | |  |
| CLDN15 | | CMKLR1 | | HYDIN | | GAS2 | |  | |  |
| PLCB1 | | GM38357 | | GM45752 | | RPS26-PS1 | |  | |  |
| P2RX1 | | CCDC152 | | GM24727 | | LGR4 | |  | |  |
| GM46637 | | G0S2 | | GM45548 | | GM15186 | |  | |  |
| MST1R | | AA414768 | | MAG | | SLC25A18 | |  | |  |
| STXBP6 | | TNFSF13 | | TEDDM2 | | ANKS1B | |  | |  |
| GM10384 | | MAP3K15 | | PTPRK | | GM16068 | |  | |  |
| GM12589 | | RFLNB | | AXDND1 | | GM10997 | |  | |  |
| MS4A8A | | BAMBI-PS1 | | OTOF | | SCARNA13 | |  | |  |
| SLC5A11 | | CREG2 | | THSD4 | | GRIN2B | |  | |  |
| FPR2 | | NAALADL1 | | GM46411 | | UGT1A5 | |  | |  |
| TNFRSF8 | | ARSI | | ABCB9 | | FOXO4 | |  | |  |
| GUCA2B | | EPHA4 | | GM8251 | | A930029G22RIK | |  | |  |
| GM15931 | | CYP3A11 | | GM17745 | | PTGES | |  | |  |
| SCNN1A | | HNMT | | CLDN7 | | GM5121 | |  | |  |
| RAB44 | | FAM78B | | CUBN | | GM9514 | |  | |  |
| P2RY1 | | PDGFRB | | CACNA1F | | COL7A1 | |  | |  |
| ID1 | | MGAT4F | | GM45053 | | FKBP9 | |  | |  |
| ZG16 | | MCF2L | | PKD1L2 | | GM47508 | |  | |  |
| B230208H11RIK | | GM16287 | | GM12346 | | GM49802 | |  | |  |
| CES2E | | NPC1L1 | | CNTN4 | | GNRH1 | |  | |  |
| ALDOB | | RNF128 | | CELF3 | | GM20033 | |  | |  |
| FABP1 | | GM16139 | | GM20692 | | GM42829 | |  | |  |
| WNT6 | | GM11707 | | FUOM | | GM48529 | |  | |  |
| COLEC12 | | INSYN2A | | EXPH5 | | GM12758 | |  | |  |
| RILP | | H2AC6 | | PMEL | |  | |  | |  |
| CITED4 | | GM44861 | | CPA6 | |  | |  | |  |
| GPSM2 | | ZFP541 | | MYT1L | |  | |  | |  |
| RHOU | | FAM187B | | CTNNA3 | |  | |  | |  |
| D930028M14RIK | | TNFSF12 | | GM12689 | |  | |  | |  |
| CDHR2 | | WIPI1 | | FOXP2 | |  | |  | |  |
| E230016K23RIK | | ENPP3 | | DOK2 | |  | |  | |  |
| GM18068 | | FCGBP | | MUC6 | |  | |  | |  |
| CYP3A13 | | 1700001K23RIK | | VDR | |  | |  | |  |

| **Supplementary Table S2 (cont.)** | | |  |  |  |  |
| --- | --- | --- | --- | --- | --- | --- |
| **Dataset of genes differentially expressed between monocytes and dendritic cells** | | | | | |  |
| **obtained from Immgen (www.immgen.org)** | | | |  |  |  |
|  |  |  |  |  |  |  |
| ***Upreg DC_vs_Mo*** | |  |  |  |  |  |
| CCR7 | LTA | SDR39U1 | NPHP1 | PLCD3 | ELMO3 | GM16305 |
| APOL7C | TTC39A | GM45051 | BFSP2 | SMIM22 | E430024P14RIK | GM37829 |
| CD4 | PPP1R16B | CTSW | GM42047 | RAD51AP1 | 9330136K24RIK | 5730405O15RIK |
| SERPINB6B | FADS1 | IL2RB | 6430562O15RIK | GGACT | GM50087 | MT-TP |
| GM8221 | BCL7A | SRC | GM37534 | GM20274 | ADGRL1 | ZFR2 |
| SIGLECH | CD72 | LZTS1 | GM3604 | GM15232 | BEND3 | C230062I16RIK |
| GM34680 | RNF180 | GM49579 | MCOLN2 | EFCAB7 | GM26520 | GM12229 |
| GPR4 | PRNP | GM48231 | TSPYL4 | 2810029C07RIK | GM44975 | CCDC62 |
| ADAM11 | FCNA | UNG | NPL | ALPK2 | 4930426L09RIK | BC051142 |
| PRSS30 | MYB | CTLA2A | OLFR755-PS1 | VEGFB | GM4924 | GM9085 |
| VCAM1 | SLC25A29 | FOXP4 | 5830444F18RIK | GM12522 | TNK2 | GM8172 |
| ENPP5 | ST8SIA1 | MRAS | SLC39A14 | P4HTM | GTF2IRD1 | LIME1 |
| GPM6B | ACTN1 | 3300005D01RIK | ZFP607B | CLSPN | GM49474 | CAMK2B |
| KIT | RASIP1 | CBARP | CAR2 | PLEKHA7 | GM18553 | CBX2 |
| CD200 | IGHD | GM37886 | C030005K06RIK | XLR4A | GM43693 | ITGB1BP2 |
| SPIB | SYNPO2 | BSN | AP1S3 | TMIGD3 | GM50230 | FBXL19 |
| SNX22 | ICOS | GM37265 | JCHAIN | CENPM | 0610040B10RIK | GM37321 |
| LAG3 | PHGDH | GM45203 | GM15283 | GM43544 | GM12592 |  |
| MYCL | ARL6 | BASP1 | TM4SF5 | GM18821 | GM48586 |  |
| MREG | TRP53I11 | CLEC1B | EPHX1 | MORN1 | GM44559 |  |
| CACNA1E | GM20429 | GM2065 | BDH1 | GM42572 | GM15550 |  |
| N4BP3 | ZFP783 | CHST3 | 5830408C22RIK | DLGAP5 | 1810014B01RIK |  |
| PROCR | MYZAP | OSBPL5 | DHRS13 | GM42508 | WDR60 |  |
| GPR157 | ARHGAP32 | MTERF2 | NCR1 | GM37879 | 4732416N19RIK |  |
| FSCN1 | SEMA7A | SERPINA3F | LCK | TRIM6 | NAF1 |  |
| P2RY10 | ATP1B1 | CHRNB1 | MYLPF | A930012O16RIK | 4930405O22RIK |  |
| CCR9 | PRR5 | SPACA6 | PPP3CC | GIMAP3 | GM15518 |  |
| CD38 | RCN1 | CHST11 | E430014B02RIK | 1810021B22RIK | GM45251 |  |
| H2-EB2 | XCR1 | EYA1 | RAB11FIP3 | DSEL | FGF2 |  |
| C1QA | PDGFA | SEMA6D | SCRN2 | HEXIM2 | ANXA9 |  |
| SLC4A8 | ZCCHC18 | GM48627 | STKLD1 | PCX | GM9316 |  |
| CD300C | GM15228 | UST | ZPBP | GM42876 | GM6789 |  |
| ASPRV1 | GABBR1 | CHIL5 | STAB2 | GM45745 | GM47283 |  |
| IL7R | GM48718 | CEP170B | CYSTM1 | ZFP808 | GM47644 |  |
| TNS1 | A530064N14RIK | MPZL1 | RAB37 | GM11110 | GM43024 |  |
| GPR68 | TENT5C | GM13543 | CLU | GM28112 | RASL2-9 |  |
| LRP8 | SPSB1 | FCER2A | GM44695 | CISH | GM15133 |  |
| MS4A1 | GM37215 | GLCCI1 | CELSR1 | PTPN21 | SAV1 |  |
| TLR12 | CXXC5 | GM37248 | SEPTIN4 | MVB12B | GM48062 |  |
| PAKAP | MSANTD1 | RNF227 | NBL1 | GM42726 | GM40466 |  |
| RTL8C | SCN2B | GM7240 | FOXM1 | NOXRED1 | GM33882 |  |
| BHLHB9 | SLC7A5 | CD5 | KCTD14 | GM37105 | 2810414N06RIK |  |
| C1QC | B230217C12RIK | MNS1 | SSX2IP | GM17088 | NXN |  |
| SNN | TNFRSF18 | PCBP4 | GM12454 | CRPPA | GM26770 |  |
| CLEC4G | NFE2L3 | GM42986 | CD5L | CACNB4 | GM45607 |  |
| PLA2G4F | GM26947 | GM36931 | RRAGB | GTSE1 | GM12185 |  |
| MAGED1 | IGLC1 | B3GALNT1 | GM14636 | GEMIN8 | AI480526 |  |
| CXCR3 | BC051537 | PTPN3 | GM45188 | GM26873 | GM43692 |  |
| CHPF | GM43145 | GM42549 | KCNA2 | BC043934 | GM44777 |  |
| SEPTIN1 | PGLYRP2 | GM4258 | C130026I21RIK | MIR5128 | GM4890 |  |
| ELDR | GM13561 | GM20008 | FBXO44 | SFXN4 | SUV39H2 |  |
| GALNT12 | IL9R | COL23A1 | GM37212 | OLFR756-PS1 | GM13665 |  |
| GEM | HRH1 | DAB2IP | MID1 | GM37470 | GM48624 |  |
| RAB30 | SRL | GM35315 | L2HGDH | GM37726 | GM50107 |  |
| TBC1D19 | GM7265 | EHHADH | HDAC11 | RPS15A-PS6 | GM15712 |  |
| HOXA7 | RAD51B | ZNRD1AS | GM10644 | CLCN2 | GM46209 |  |
| LMNTD2 | SLC12A3 | NME4 | 3830408C21RIK | GM18990 | NDUFS5-PS |  |

**A**


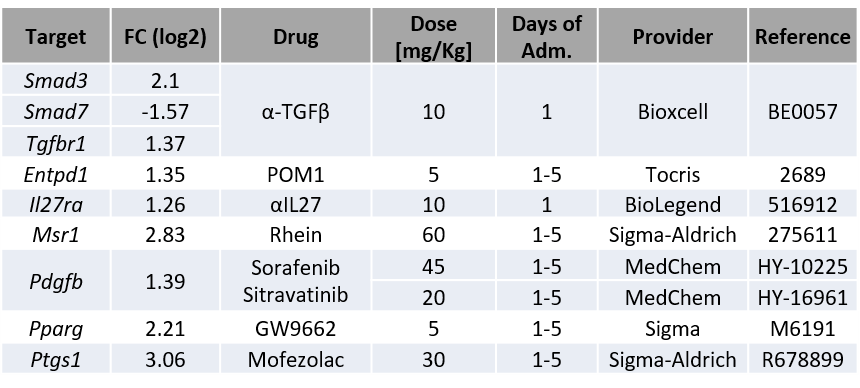

**B**

**Supplementary Figure S1: Combination of vaccine with inhibitors does not improve vaccine immunogenicity.** (A) List of genes differentially expressed in DC-IL-10^+^ with their corresponding fold-change (FC), and the inhibitor used, dose and days of administration. (B) C57BL/6J mice (n=4-8/group) were immunized with OVA + Imiquimod vaccine with selected inhibitors or control (vehicle or isotype antibodies) with doses described in A. One week later animals were sacrificed and responses against peptide OVA(257-264) were determined by IFN-gamma ELISPOT in each mouse, measuring the number of spot forming cells (SFC). Data (mean + SEM) have been normalized according to mean values obtained in control group. (*, P<0.05)

**Enrichment score (ES)**

**Supplementary Figure S2: DC-IL10^+^ have a monocytic origin.** Gene set enrichment analysis of genes found in DC-IL10^+^ using a gene signature of an immunosuppressive monocyte-derived DC population described in mice chronically infected with LCMV (Cunningham CR, et al. PLoS Pathog. 2016;12:e1005356). Statistical parameters (P and FDR) were provided by the GSEA webpage, calculated as described in Subramanian et al, *Proc Natl Acad Sci U S A* (2005) 102:15545–15550. doi: 10.1073/PNAS.0506580102.


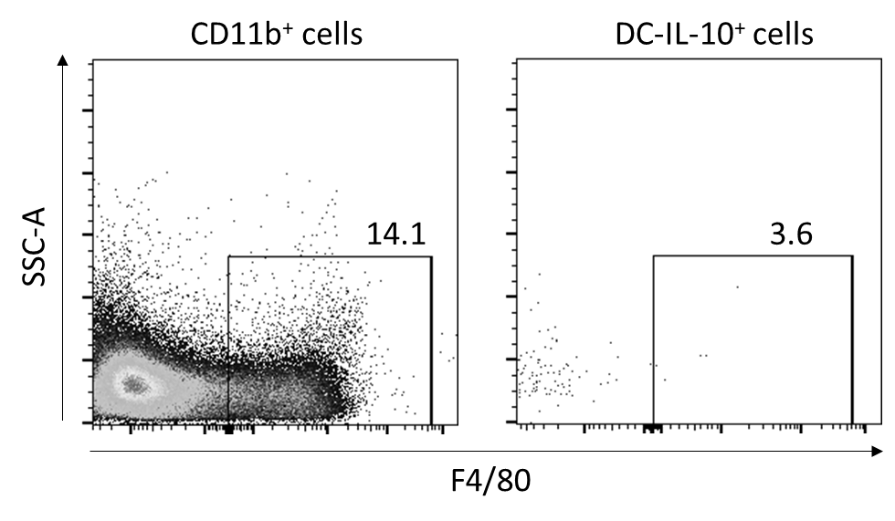


**Supplementary Figure S3: DC-IL10^+^ do not express F4/80.** Vert-X mice were immunized with OVA + Imiquimod and 2 days later splenocytes were analyzed by flow cytometry. CD11b^+^ cells (left panel) or DC-IL-10^+^ cells (CD11c^high^, I-A^b^ ^high^, GFP^+^; right panel), were gated from total splenocytes, and expression of F4/80 was analyzed in each cell subset. Representative plots are shown.


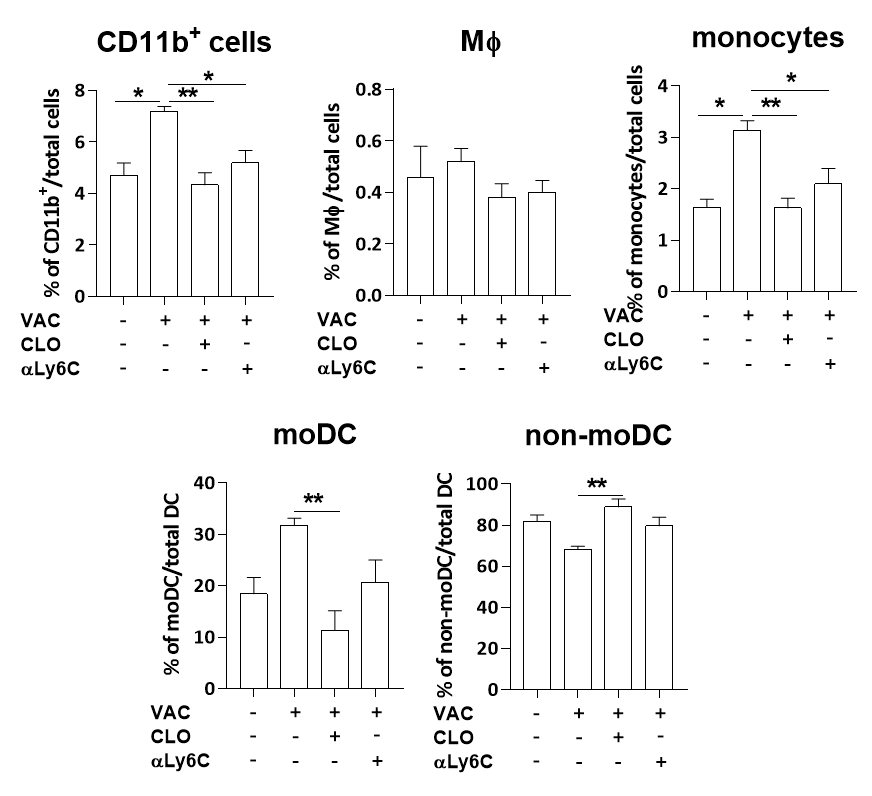


**Supplementary Figure S4: Effect of clodronate and antiLy6C antibodies on different myeloid cell subsets.** Vert-X mice (n=4 mice/group) were immunized with OVA + Imiquimod (VAC) with or without depleting agents: clodronate (CLO) or anti-Ly6C antibodies (αLy6C). The percentage of CD11b^+^ cells, macrophages (Mφ, defined as CD11b^+^ F4/80^+^ cells and monocytes (CD11b^+^, F4/80^-^ and Ly6C^+^ cells) was calculated for each group. Similarly, in total DC, gated as CD11c^high^, I-A^b^ ^high^ cells, the percentage of monocyte-derived DC (defined as CD64^+^ Ly6C^+^) and non-monocyte-derived DC was calculated.

**A**


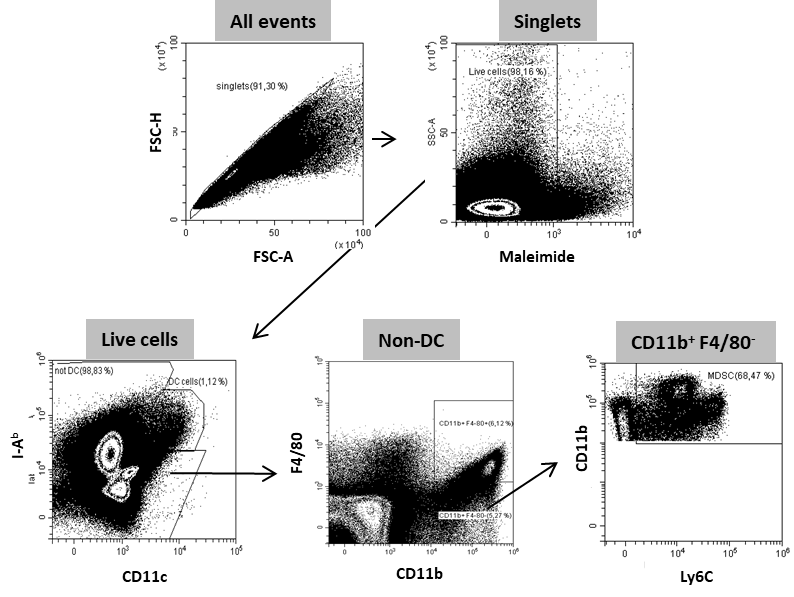


**B**

**Supplementary Figure S5: Characterization of LLC-OVA cells and tumors.** (A) C57BL/6J mice (n=4) were immunized with OVA + Imiquimod. One week later responses were evaluated by ELISPOT using 8 x 10^5^ splenocytes and irradiated LLC-OVA and parental LLC cells (8 x 10^4^ cells/well) (****; P<0.0001). (B) Spleens of mice with LLC-OVA tumors were homogenized and the proportion of MDSC was determined by flow cytometry. Gating strategy is shown.

**B**

**A**


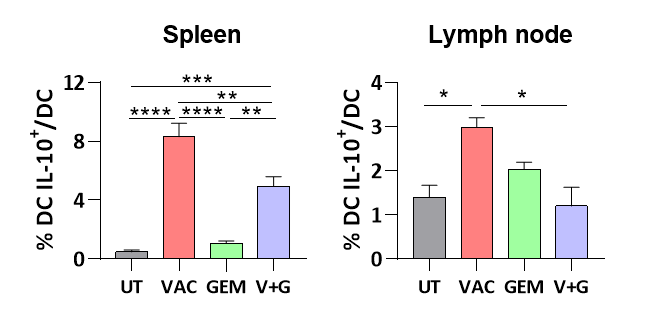


**Supplementary Figure S6: Gemcitabine decreases the percentage of DC-IL-10^+^ induced by vaccination in mice with LLC-OVA tumors.** (A) Vert-X mice with 5-6 mm LLC-OVA tumors (n=4/group) were left untreated (UT) or treated with OVA + Imiquimod vaccine (VAC), Gemcitabine (GEM) or the combination (V+G). Two days after vaccination they were sacrificed and the percentage of DC IL-10^+^ was determined in the spleen (A) or in tumor-draining lymph nodes (A). (*, P<0.05; **, P<0.01; ***, P<0.001;****, P<0.0001).

**UT**

**VAC**


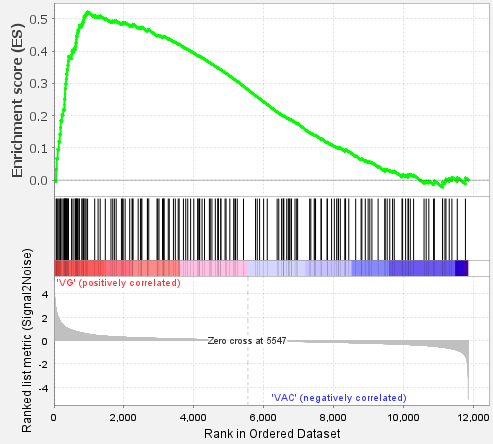

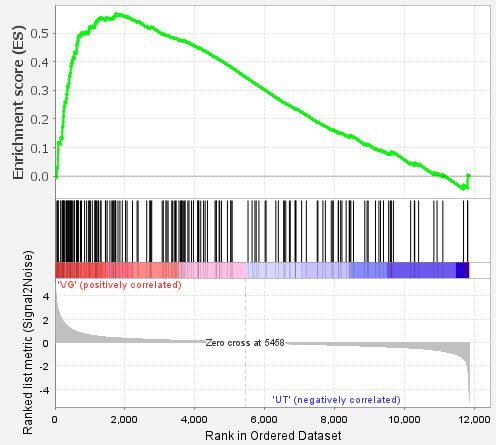


NES = 1.83

*P* <0.001

FDR = 0.004

NES = 2.08

*P* <0.001

FDR <0.001

**GEM**


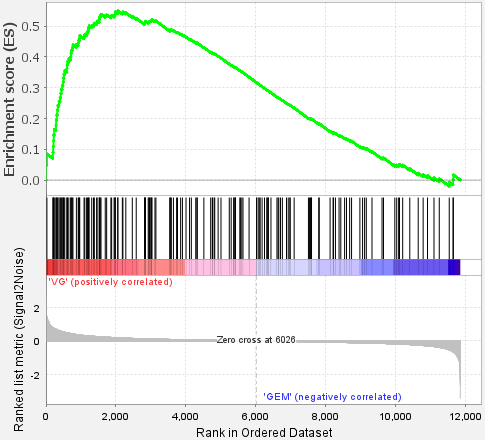


NES = 1.98

*P* <0.001

FDR <0.001

**Supplementary Figure S7: Gene set enrichment analysis (GSEA) of the Vaccine + gemcitabine group using the “Allograft rejection” GSEA hallmark.** Genes expressed in the Vaccine + gemcitabine group were compared with those genes of untreated (UT), Vaccine (VAC) or gemcitabine (GEM) monotherapy groups, and these data were used for gene set enrichment analyses using the “Allograft rejection” hallmark. Statistical parameters (P and FDR) were provided by the GSEA webpage, calculated as described in Subramanian et al, *Proc Natl Acad Sci U S A* (2005) 102:15545–15550. doi: 10.1073/PNAS.0506580102.


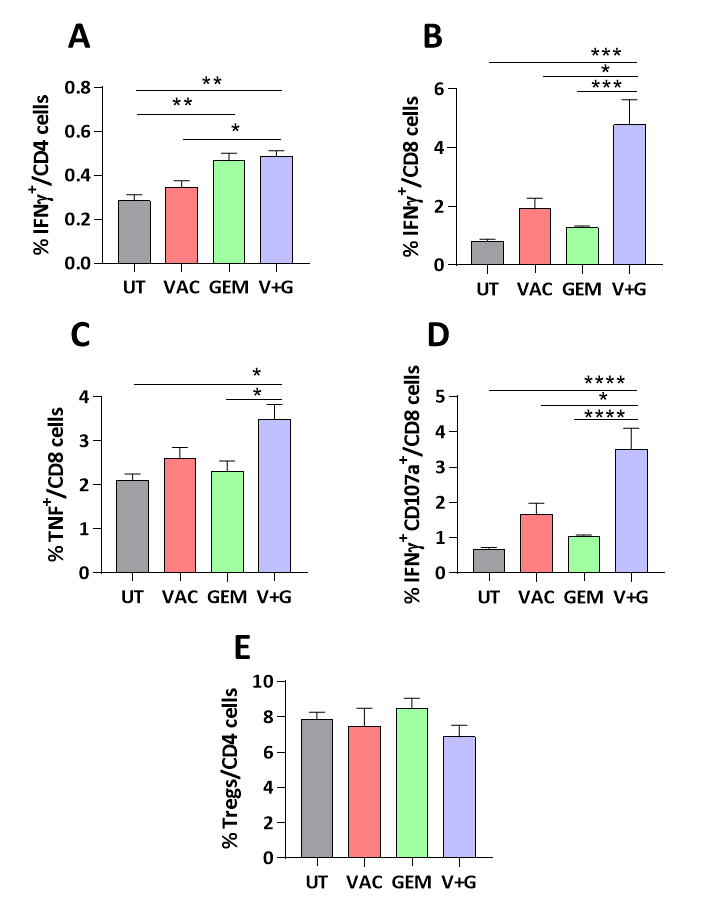


**Supplementary Figure S8: Enhanced T cell effector functions in the spleen of mice treated with Vaccine + gemcitabine.** C57BL/6J mice bearing 5-6 mm LLC-OVA tumors (n=5-9 mice/group) were untreated (UT) or treated with OVA + Imiquimod vaccine (VAC), Gemcitabine (GEM) or the combination (V+G). One week later they were sacrificed and splenocytes were stimulated with CD4 epitope OVA(323-339) (A) or CD8 epitope OVA(257-264) (B-D) to analyze IFN-gamma, TNF or IFN-gamma/CD107a expression by T cells. (E) Percentage of Tregs in unstimulated splenocytes was also determined. (*, P<0.05; **, P<0.01; ***, P<0.001;****, P<0.0001).


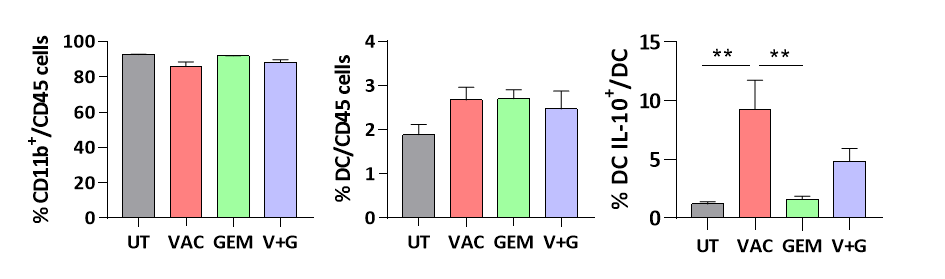


**C**

**B**

**A**

**Supplementary Figure S9: Effect of the Vaccine + gemcitabine combination in tumor myeloid cells.** C57BL/6J mice bearing 5-6 mm LLC-OVA tumors (n=5-9 mice/group) were untreated (UT) or treated with OVA + Imiquimod vaccine (VAC), Gemcitabine (GEM) or the combination (V+G). One week later they were sacrificed and percentages of CD11b^+^ cells (A) and DC, defined as CD11c^high^, I-A^b^ ^high^ cells (B) within the CD45 subset were determined by flow cytometry. (C) Vert-X mice (n=4/group) with LLC-OVA tumors were treated as above. Two days after treatment the percentage of DC IL-10^+^ was determined by flow cytometry.


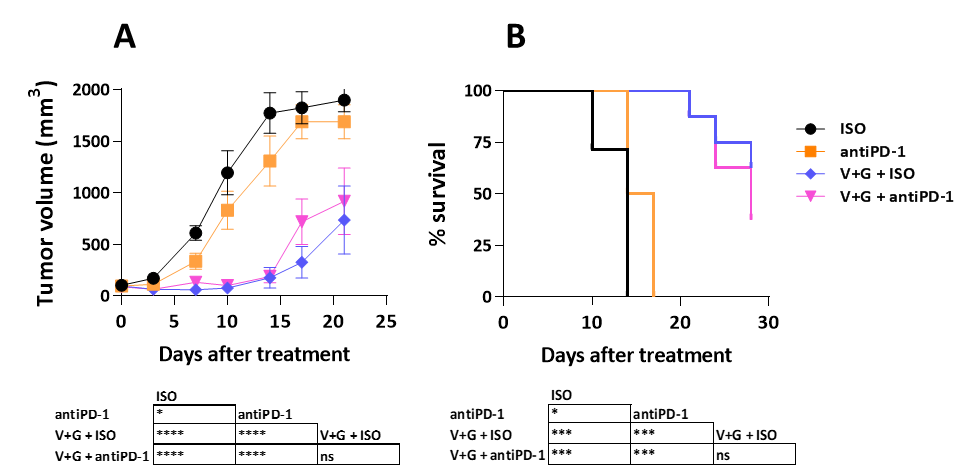


**Supplementary Figure S10: Higher doses of ant-PD-1 did not improve the therapeutic effect when combined with vaccine + gemcitabine in LLC-OVA tumors.** C57BL/6J mice bearing 5-6 mm LLC-OVA tumors (n=8 mice/group) were treated with isotype control antibody (ISO), anti-PD-1 (in both cases using 200 μg per administration), vaccine + gemcitabine with isotype control antibody (V+G + ISO) or with anti-PD-1 (V+G + antiPD-1). Tumor growth (A) and animal survival (B) were determined twice per week. (*, P<0.05; ***, P<0.001;****, P<0.0001).

**Supplementary Figure S11. Higher *in vitro* sensitivity to gemcitabine of LLC-OVA vs B16-OVA.** LLC-OVA and B16-OVA cells ( 1.5 x 10^4^ cells/well) were cultured *in vitro* with increasing doses of gemcitabine. Two days later the number of viable cells was counted. IC_50_ was determined by using the GraphPad Prism software after plotting Gemcitabine concentrations vs the number of cells.

**Supplementary Figure S12. Higher proportion of infiltrating Tregs in B16-OVA vs LLC-OVA tumors.** Five mm LLC-OVA or B16-OVA tumors (n=5-6/group) were obtained from untreated C57BL/6J mice and flow cytometry was used to determine the proportion of Tregs. (****; P<0.0001)
